# A survey of Preventive Measures Used and their Impact on Central Line-Associated Bloodstream Infections (CLABSI) in Intensive Care Units (SPIN-BACC)

**DOI:** 10.1186/1471-2334-13-562

**Published:** 2013-12-01

**Authors:** Milagros Gonzales, Isabelle Rocher, Élise Fortin, Patricia Fontela, Mohammed Kaouache, Claude Tremblay, Charles Frenette, Caroline Quach

**Affiliations:** 1 Department of Pediatrics, The Montreal Children’s Hospital, McGill University, Quebec, Québec, Canada; 2 Institut National de Santé Publique du Québec, Québec and Montréal, Montréal, Québec, Canada; 3 Department of Epidemiology, Biostatistics, and Occupational Health, McGill University, Montreal, Québec, Canada; 4 Centre Hospitalier Universitaire de Québec - Pavillon Hôtel-Dieu de Québec, Québec, Québec, Canada; 5 Department of Medical Microbiology and Infectious Diseases, McGill University Health Center, McGill University, Montreal, Québec, Canada

**Keywords:** Central line associated bloodstream infections, Intensive care units, Preventive measures, Bundles, Compliance

## Abstract

**Background:**

The Quebec central line-associated bloodstream infections (CLABSI) in intensive care units (ICUs) Surveillance Program saw a decrease in CLABSI rates in most ICUs. Given the surveillance trends observed in recent years, we aimed to determine what preventive measures have been implemented, if compliance to measures was monitored and its impact on CLABSI incidence rates.

**Methods:**

All hospitals participating in the Quebec healthcare-associated infections surveillance program (SPIN-BACC – n = 48) received a 77-question survey about preventive measures implemented and monitored in their ICU. The questionnaire was validated for construct, content, face validity, and reliability. We used Poisson regression to measure the association between compliance monitoring to preventive measures and CLABSI rates.

**Results:**

Forty-two (88%) eligible hospitals completed the survey. Two components from the maximum barrier precautions were used less optimally: cap (88%) and full sterile body drape (71%). Preventive measures reported included daily review of catheter need (79%) and evaluation of insertion site for the presence of inflammation (90%). Two hospitals rewired lines even if an infection was suspected or documented.

In adult ICUs, there was a statistically significant greater decrease in CLABSI rates in ICUs that monitored compliance to preventive insertion measures, after adjusting for teaching status and the number of hospital beds (p = 0.036).

**Conclusions:**

Hospitals participating to the SPIN-BACC program follow recommendations for CLABSI prevention, but only a minority locally monitor their application. Compliance monitoring of preventive measures for catheter insertion was associated with a decrease in CLABSI incidence rates.

## Background

A surveillance program for central line-associated bloodstream infections (CLABSI) in intensive care units (ICUs) was implemented in the province of Quebec, Canada in 2003 under the purview of the Surveillance provinciale des Infections Nosocomiales (SPIN) network, part of the Institut national de Santé publique du Québec (INSPQ). Hospitals are required to report monthly data on CLABSI incidence, year-round. Surveillance results are published annually (http://www.inspq.qc.ca) and are used for benchmarking [1]. CLABSI incidence rates decreased from 2007 – at the onset of the mandatory surveillance program – to 2012–13, for all ICU types except neonatal ICUs [2].

The implementation of bundles, a set of evidence-based preventive measures that when applied together have been shown to decrease healthcare associated infections, has previously been described as a mean to reduce CLABSI rates [3-8]. Given the surveillance trends observed in recent years, we aimed to determine what preventive measures have been implemented in Quebec ICUs, determine if compliance to these preventive measures was monitored and understand the impact of compliance monitoring on CLABSI incidence rates.

## Methods

### Study setting and population

A voluntary survey was sent to all SPIN-BACC participating hospitals. Since 2007, participation to the surveillance program is mandatory for all ICUs with at least 10 beds and encouraged for others. SPIN implementation, definitions and methods have been previously described [1,9]. CLABSI definitions used are based on the 2008 National Healthcare Safety Network definitions [10].

### Study design

A 77-question, cross-sectional survey, based on recommendations from the Centers for Disease Control and Prevention (CDC) guidelines [11] and the “Safer Healthcare Now!” campaign from the Canadian Patient Safety Institute [12] was developed, validated and piloted by SPIN members and the Quebec Nosocomial Infections Committee (CINQ) through focus groups for construct, content, face validity, and reliability. The survey was divided in five sections: 1) infection control and prevention resources, 2) infection surveillance in the ICU, 3) local infection control and prevention program, 4) use of bundles in the ICU and 5) miscellaneous. Survey questions types included multiple choices, checkboxes, and short open-ended answers. CLABSI incidence rates for each ICU were available through the SPIN-BACC program from April 2007 to March 2013.

The outcome of interest was change in pooled annual CLABSI rates from baseline (first year of participation in SPIN-BACC surveillance program between April 1^st^ 2007 and March 31^st^ 2010) to the post-survey rate (surveillance year April 1^st^ 2012 to March 31^st^ 2013). The study was approved by the McGill University research ethics board. Compliance was defined as healthcare workers’ adherence to recommended measures to reduce CLABSI rates adopted by the ICU. Monitoring referred to active auditing of compliance.

### Logistics

An online survey, designed using the FluidSurveys® software (URL: http://www.fluidsurveys.com) was sent to infection preventionists using automated personalized email invitations. Surveys were sent in December 2011 with reminders in January and February 2012. Survey data collection was finalized by March 2012.

### Analysis

Descriptive statistics were used to summarize variables. Poisson regression was used to estimate the change in infection rates per 1000 central venous catheter (CVC)-days from baseline to 2012–2013 (post-survey). Analyses were restricted to the following variables due to their reported evidence-based impact on infection rates: hand hygiene, use of cap, mask, sterile gown, sterile gloves, full body drape, daily review of CVC need, and regular evaluation of the CVC insertion site. Hospitals that did not answer questions regarding compliance monitoring were considered as not monitoring compliance.

Compliance monitoring was included in the model as a binary variable but also as an interaction term with time period. This was used to estimate the effect of compliance monitoring on the evolution of rates from the reference period to 2012–2013. Only ICUs that participated to SPIN-BACC for at least one complete year between 2007 and 2010 were included in our statistical analyses. A subgroup analysis was performed to estimate the effect of compliance monitoring in adult (teaching and non-teaching), pediatric (PICU), and neonatal (NICU) ICUs. Variables with a p-value < 0.1 in the univariate analysis were kept for the multivariate analysis. Multivariate models were adjusted for the hospital teaching status and the number of hospital beds. Statistical analysis was conducted using the PROC GENMOD procedure in SAS v9.2 (NC, USA).

## Results

### Preventive measures implemented at the hospital level (n = 42)

#### Description of participating centers

Forty-two of 48 eligible hospitals, representing 47 adult ICUs, 3 PICUs, and 5 NICUs, completed the survey (response rate 87.5%). Three surveys where only hospital’s characteristics questions were answered (<25% of survey completed) were excluded. All non-participating hospitals were non-teaching. Basic description, pooled CLABSI rates (baseline and post-survey year) and device utilization ratios for participating units, stratified by ICU type, are presented in Table 1. A significant decrease in CLABSI rates was observed in teaching adult ICUs where rates decreased from 1.51 to 0.85/1000 CVC-days (p = 0.0001).

**Table 1:** Description and summary of pooled annual CLABSI rates (as reported to the SPIN-BACC program) in participating ICUs from 2007 to 2013

**ICU type**	**ICU (n)**	**ICU beds *Median (interquartile range)***	**Pooled mean rates/1000 CVC-days (95% CI)**		**Device utilization ratios (95% CI)**	
			**Baseline**	**Post-survey period**	**Baseline**	**Post-survey period**
Adult non-teaching						
Combined*	17	10 (8 – 11)	1.71 (1.09-2.54)	1.15 (0.71-1.78)	0.295 (0.291-0.299)	0.356 (0.352-0.360)
Adult teaching						
Burn	2	10	1.51 (1.25-1.81)	0.85 (0.66-1.08)	0.605 (0.602-0.608)	0.590 (0.587-0.592)
Cardiac	3	9 (7 – 9.5)				
Combined*	20	16 (11.5 – 21)				
Medical	2	11.5 (9.25 – 13.75)				
Surgical	3	20 (17 – 22)				
PICU	3	12 (9.5 – 18)	2.31 (1.19-4.03)	2.58 (1.50-4.13)	0.209 (0.205-0.212)	0.239 (0.235-0.242)
NICU	5	26 (20.75 – 29)	4.57 (3.35-6.10)	6.19 (4.95-7.66)	0.603 (0.592-0.613)	0.616 (0.607-0.626)

ICU: Intensive Care Unit; CVC: Central venous catheter: PICU: pediatric ICU; NICU: Neonatal ICU.

CI: Confidence interval.

*Combined: medical and surgical or cardiac.

Baseline refers to the first consecutive year of participation between April 1^st^ 2007 and March 31^st^ 2010.

Post-survey period refers to the consecutive year of participation between April 1^st^ 2012 and March 31^st^ 2013.

#### CVC insertion preventive measures

Two components were used less optimally in responding hospitals: wearing of cap (n = 37, 88%) and use of full sterile body drape (n = 30, 71%). All sites reported using a 2% chlorhexidine in 70% alcohol solution for skin antisepsis prior to CVC insertion for patients aged 2 months and over. Ultrasound guidance for CVC placement was used in 35 hospitals (83%). In adult ICUs, instructions for optimal CVC site selection were reported in only 24 centers; preference was given to the subclavian (n = 13, 54%), jugular (n = 3, 13%), either (n = 4, 17%), or other vein (n = 1, 4%), while 3 (13%) reported no preference.

#### CVC maintenance preventive measures

Access hubs were disinfected with either alcohol (n = 13, 31%), aqueous chlorhexidine (n = 3, 7%), or a 2% chlorhexidine 2% - 70% alcohol solution (n = 8, 19%) and scrubbed for 5 (n = 2), 10 (n = 3), 15 (n = 10) or 30 seconds (n = 12). Daily review of CVC need was performed in 33 hospitals (79%). Insertion site was evaluated for the presence of inflammation either daily (n = 22, 52%) or with each dressing change (n = 9, 21%). Most hospitals had exclusively dedicated lines for hemodialysis and hemofiltration (n = 33, 79%), or total parenteral nutrition (TPN) (n = 34, 81%). Routine use of CVC locks was reported in 19 institutions (45%), while 12 (29%) restricted their use to certain patient population. Citrate was mostly used for patients on hemodialysis. Only 7 hospitals reported using antibiotic locks.

#### Dressing and intravenous (IV) tubing

The use of sterile gauze alone for the initial dressing was privileged in 5 teaching hospitals. Transparent semi-permeable dressings were used by most, either alone (initial: 14; subsequent: 9) or combined with sterile gauze when the CVC insertion site was leaking (initial: 23; subsequent: 33). Some hospitals (n = 8, 19%) used sponges or other chlorhexidine dressings; either for all patients (n = 5), hemodialysis (n = 2) or oncology patients (n = 1). IV tubing replacement periods were respected in most hospitals: after ≤ 96 hours of routine use (n = 41, 98%), after ≤24 hours when used for transfusion (n = 38, 90%), and after 24 hours if used for TPN (n = 35, 83%). Two hospitals reported rewiring lines despite suspicion or documentation of an infection.

#### Bundles

Most hospitals implemented insertion (n = 26) and maintenance (n = 25) bundled practices [12]. Checklists for insertion measures were used in 19 institutions (73%), of which 16 were teaching hospitals (p = 0.01). The checklist was filled by a nurse assisting the procedure or was a self-evaluation (n = 8, 42% for each).

#### Needleless device use

Needleless devices were used in all but 2 hospitals (95%). Negative displacement mechanical valve systems (n = 19, 45%) were mostly used, followed by split septum connectors (n = 15, 36%) and positive displacement mechanical valves (n = 2, 5%). Four facilities used more than one type of connector. Only 8 sites (19%) reported using antiseptic coated-catheters: at all times (n = 5) or restricted to certain patient populations: hematology/oncology (n = 1), prolonged TPN use (n = 1), patients with recurrent line sepsis (n = 2) and burned patients (n = 1).

### Preventive measures implemented at the ICU level (n = 55)

#### Compliance to preventive measures

**Figure 1:**
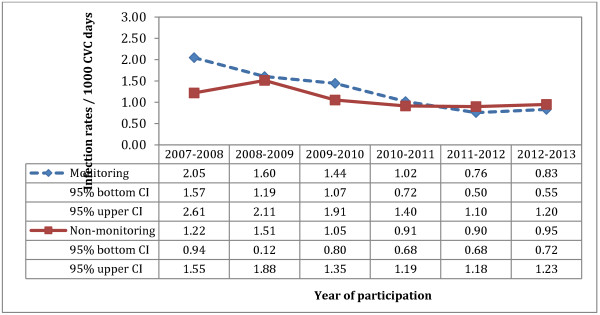
Variation in pooled CLABSI rates of participating adult ICUs according to monitoring of compliance to hand hygiene, use of mask, sterile gloves and sterile gown.

Less than 50% of hospitals reported monitoring compliance to recommended measures. In ICUs where compliance was measured, the median compliance was >75%. One ICU had not participated to SPIN-BACC during the baseline period and was excluded from the analyses. Because they were highly correlated, the following 4 CVC insertion preventive measures were combined: hand hygiene, use of mask, gloves and gown donning. Forty-five adult ICUs had implemented these 4 measures: 13 monitored compliance while 32 did not. After adjusting for the number of hospital beds and teaching status, the effect of compliance monitoring for CVC insertion measures remained significantly associated with a greater decrease in CLABSI rates (p = 0.036). The daily review of CVC need and the inspection of CVC insertion site were strongly correlated with the preventive measures for CVC insertion; we were therefore unable to assess their independent impact on CLABSI incidence. Figure 1 illustrates annual CLABSI rates with 95% confidence intervals (CI) in adult ICUs, stratified by compliance monitoring for the insertion preventive measures. Units that monitored compliance to the four combined measures had overall higher CLABSI incidence rates in 2007–2008 (n = 13, IR: 2.05; 95% CI 1.57 – 2.61), at the outset of the mandatory surveillance program, compared to ICUs that did not monitor compliance (n = 32, IR: 1.22; 95% CI 0.94 – 1.55).

Table 2 details the association between monitoring of compliance and CLABSI incidence rate ratios (baseline to post-survey period) in adult ICUs. In the univariate analysis, ICUs that monitored compliance to hand hygiene/use of sterile gloves, to wearing masks and gowns and to the use of sterile drapes saw a greater CLABSI rate decrease compared to units that did not monitor compliance. When all ICU types were combined, there was a trend toward a greater decrease in CLABSI rates in ICU that monitored (incidence rate ratio baseline vs. 2012–13 period: 0.496) compared to ICUs that did not monitor compliance to evaluation at CVC site (IRR: 0.735). This difference was however not statistically significant (p = 0.103). In NICUs, units that monitored compliance to the evaluation of CVC insertion site had a trend toward a greater decrease in CLABSI rates (p = 0.18).

**Table 2:** Univariate analysis of preventive measures and pooled annual CLABSI rates in adult ICUs

**Compliance monitored for:**	**Number of units**	Rate* (baseline)§	Rate* (post-survey)	**∆ rates**	**IRR**	**p-value**
Hand hygiene and sterile gloves						
No	33	1.30	0.95	−0.35	0.73	0.041
Yes	13	1.98	0.83	−1.15	0.42	
Cap						
No	28	1.35	0.98	−0.37	0.73	0.051
Yes	13	1.98	0.83	−1.15	0.42	
Mask and sterile gown						
No	32	1.28	0.95	−0.33	0.74	0.040
Yes	13	1.98	0.83	−1.15	0.42	
Sterile full body drape						
No	20	1.47	1.11	−0.36	0.76	0.046
Yes	13	1.98	0.83	−1.15	0.42	
Daily review of CVC need						
No	23	1.18	0.86	−0.32	0.73	0.183
Yes	13	2.02	0.94	−1.08	0.47	
Evaluation of CVC insertion site						
No	31	1.33	0.85	−0.48	0.64	0.187
Yes	12	2.00	0.87	−1.13	0.44	

*Rate: /1,000 CVC-days; CVC: central venous catheter; IRR: Incidence rate ratio.

§ Baseline: the first year of ICU participation to the SPIN program between 2007 and 2010.

## Discussion

Compliance monitoring of implemented recommended measures to prevent CLABSI is not yet widespread in Quebec ICUs. Participation to our survey was excellent and showed that the majority of hospitals have implemented measures to prevent CLABSI. Only two thirds of hospitals have implemented insertion and maintenance bundles, and less than half monitor compliance to preventive measures, despite their reported effectiveness in decreasing CLABSI incidence rates [3-8]. Our study showed that ICUs where compliance monitoring was implemented had a greater decrease in their CLABSI rates compared to ICUs that did not monitor compliance to measures [5]. The association found between compliance monitoring and CLABSI rates decrease does not however imply causality. Moreover, it is possible that ICUs with higher CLABSI rates at the outset of the SPIN-BACC surveillance program were prompted to implement all recommended preventive measures. In adult ICUs, compliance monitoring of all components of the maximum barrier precautions was associated with a greater decrease in CLABSI rates.

Among the measures less applied, the use of full body drape during CVC insertion should be reinforced [11,13]. The use of a ready-to-use kit for CVC insertion, also containing a full body size drape, should improve compliance, as it would no longer rely on the operator’s judgement. The implementation of daily assessment of CVC need is simple, requires little resource and should thus be reinforced [14].

Although most hospitals followed the minimum 96-hour interval for replacement of continuously used IV administration sets, many hospitals proceeded at shorter intervals. More frequent replacement of IV tubing results in unnecessary system access and increased costs without reducing the risk of infection [15].

Because of their safety profile, needleless devices are now routinely used. However, use of some needleless devices has been associated with increased CLABSI rates [16,17]. In 2008, the Society for Healthcare Epidemiology of America published a supplement to their guidelines recommending avoiding the use of positive displacement needleless connectors [18]. Jarvis et al. reported an increase in bloodstream infections in five hospitals where split septum connectors were replaced with mechanical valves needleless devices generating either negative or positive displacement; infection rates decreased after hospitals switched back to split septum connectors [19]. In its latest recommendations, the CDC acknowledges the use of a needleless system, but privileges the use of a split septum device over mechanical valves [11]. Close monitoring of CLABSI rates in Quebec ICUs where mechanical valves are used is ongoing.

## Conclusions

This study has several limitations. First, all participating units could not be included in the statistical analysis because of missing data. Moreover, the study design was not appropriate to assess causality between compliance to measures and reduction in CLABSI incidence rates. The survey was cross-sectional and asked for the presence of various preventive measures at the time of survey. The exact implementation date of compliance monitoring to bundles is uncertain. We were thus unable to say whether compliance monitoring was initially part of the implemented intervention or if it was an added component after sites saw high infection rates in their ICUs. We did not document the frequency at which audits were carried out. It is currently unclear if these should be done all the time, for a certain proportion of CVC insertion or only for specific insertion/maintenance measures. A continuous auditing of compliance may be very resource intense and not add much if compliance is 100% for a given measure. Nevertheless, we showed that ICUs where compliance monitoring is done saw a greater decrease in their CLABSI rates. A stratified analysis could not be performed for PICUs and NICUs due to the small sample size. A more in-depth analysis should be done regarding NICUs, as their rates remain high despite the use of preventive measures. It is also possible that a social desirability bias was present in this survey results, as all questions referred to recommended measures. It is however unlikely that this bias was differential in terms of CLABSI rate outcome.

This study allowed us to better understand the use of preventive measures in Quebec ICUs, the implementation of compliance monitoring and its potential impact on CLABSI rates. Hospitals participating to the SPIN-BACC program follow most recommendations for CLABSI prevention. However, only a minority locally monitor the application of these measures. Shortage of resources, either financial or human, came out as the main cause for lack of formal and thorough surveillance in institutions.

## Abbreviations

CLABSI: Central line-associated bloodstream infection; ICU: Intensive care unit; PICU: Pediatric intensive care unit; NICU: Neonatal intensive care unit; ICP: Infection control practitioner; CVC: Central venous catheter; SPIN-BACC: Surveillance provinciale des bactériémies nosocomiales – Bactériémies associées aux cathéters centraux; INSPQ: Institut national de santé publique.

## Competing interests

All authors declare that they have no competing interests.

## Authors’ contributions

MG: Designed the survey, data collection, analyzed data and wrote first draft of manuscript; IR: Participated in survey design, data collection, and reviewed the manuscript; EF: participated in study design, data analysis and reviewed the manuscript; PF: participated in study design and reviewed the manuscript; MK: performed data analysis and reviewed the manuscript; CT: participated in survey design and reviewed the manuscript; CF: participated in survey design and reviewed the manuscript; CQ designed the study, reviewed data analysis, wrote the manuscript. All authors have reviewed and approved the final version of the manuscript.

## Pre-publication history

The pre-publication history for this paper can be accessed here:

http://www.biomedcentral.com/1471-2334/13/562/prepub
